# Gold-ionic liquid nanofluids with preferably tribological properties and thermal conductivity

**DOI:** 10.1186/1556-276X-6-259

**Published:** 2011-03-28

**Authors:** Baogang Wang, Xiaobo Wang, Wenjing Lou, Jingcheng Hao

**Affiliations:** 1 State Key Laboratory of Solid Lubrication, Lanzhou Institute of Chemical Physics, Chinese Academy of Sciences, Lanzhou 730000, China; 2 Key Laboratory of Colloid and Interface Chemistry, Shandong University, Ministry of Education, Jinan 250100, China; 3 Graduate School of Chinese Academy of Sciences, Beijing 100039, China

## Abstract

Gold/1-butyl-3-methylimidazolium hexafluorophosphate (Au/[Bmim][PF_6_]) nanofluids containing different stabilizing agents were fabricated by a facile one-step chemical reduction method, of which the nanofluids stabilized by cetyltrimethylammonium bromide (CTABr) exhibited ultrahighly thermodynamic stability. The transmission electron microscopy, UV-visible absorption, Fourier transform infrared, and X-ray photoelectron characterizations were conducted to reveal the stable mechanism. Then, the tribological properties of these ionic liquid (IL)-based gold nanofluids were first investigated in more detail. In comparison with pure [Bmim][PF_6_] and the nanofluids possessing poor stability, the nanofluids with high stability exhibited much better friction-reduction and anti-wear properties. For instance, the friction coefficient and wear volume lubricated by the nanofluid with rather low volumetric concentration (1.02 × 10^-3^%) stabilized by CTABr under 800 N are 13.8 and 45.4% lower than that of pure [Bmim][PF_6_], confirming that soft Au nanoparticles (Au NPs) also can be excellent additives for high performance lubricants especially under high loads. Moreover, the thermal conductivity (TC) of the stable nanofluids with three volumetric fraction (2.55 × 10^-4^, 5.1 × 10^-4^, and 1.02 × 10^-3^%) was also measured by a transient hot wire method as a function of temperature (33 to 81°C). The results indicate that the TC of the nanofluid (1.02 × 10^-3^%) is 13.1% higher than that of [Bmim][PF_6_] at 81°C but no obvious variation at 33°C. The conspicuously temperature-dependent and greatly enhanced TC of Au/[Bmim][PF_6_] nanofluids stabilized by CTABr could be attributed to micro-convection caused by the Brownian motion of Au NPs. Our results should open new avenues to utilize Au NPs and ILs in tribology and the high-temperature heat transfer field.

## Introduction

Gold nanoparticles (Au NPs) are always the hotspot of scientific research owing to their unique chemical and physical properties [1,2], high chemical stability and potential applications in optics, catalysts, sensors, and biology [3]. During the past several decades, a number of research groups have focused on the synthesis, characterization, properties, and applications of gold nanomaterials, and great progress in this field has been made [1,2,4-8]. To date, Au NP chemistry and physics has emerged as a broad new subdiscipline in the domain of colloids and surfaces [9]. On the other hand, ionic liquids (ILs) have also been widely studied due to their unique physicochemical properties such as negligible vapor pressure, nonflammability, high ionic conductivity, low toxicity, as good solvents for organic and inorganic molecules, high thermal stability, and wide electrochemical window [10]. Thus, ILs have attracted interests as benign solvent systems or green stabilizers for synthesizing gold nanomaterials in the past two decades [5-8]. The Brust-Schiffrin [5,7], microwave heating [11], gamma-radiation [12], sonochemical [13], seed-mediated [6], photochemical reduction [14], and electron beam irradiation [15] methods have been used to prepare gold nanomaterials in the existence of ILs, of which the Brust-Schiffrin method is most facile and popular.

The stable Au NPs in water or organic solvents have been successfully fabricated using functionalized ILs or surfactants as capping agents and their optical, electrical, catalytic, biological, and thermal properties have been widely studied [4,5,16-18]. While Au NPs synthesized in ILs are usually prone to aggregate in the absence of additional stabilizers [11,14,15], which greatly restrains their physicochemical properties and applications. Moreover, researchers have paid more attention to synthesize gold nanocrystals, while the Au/IL nanofluids may have more potential applications in various fields. Recently, Dash and Scott [7] reported that the stable Au NPs and bimetallic PdAu NPs were successfully synthesized in 1-butyl-3-methylimidazolium hexafluorophosphate ([Bmim][PF_6_]) by using NaBH_4 _as reductant and trace 1-methylimidazole as stabilizer. They found that the catalytic activity of the stable PdAu/[Bmim][PF_6_] nanofluid was remarkably higher than that of the unstable one in which the aggregation of PdAu NPs easily occurred. The pioneer work of Dash et al. indicated that the Au/IL nanofluids were expected to combine the excellent properties and open new avenues to the utilization of Au NPs and ILs. Based on this idea, we would like to make more effort on exploring the fabrication of stable Au/IL nanofluids as well as their properties.

Cetyltrimethylammonium bromide (CTABr) is a commercially available surfactant, which has been widely used as capping agent of Au NPs and shape controller of gold nanorods in aqueous systems [19]. To our best knowledge, CTABr has not yet been used as stabilizer for the synthesis of Au NPs in the ILs. In the present article, we synthesized Au NPs in [Bmim][PF_6_] using CTABr as capping agent and NaBH_4 _as reductant. The Au NPs modified by CTABr exhibit ultrahigh stability and homogeneity in [Bmim][PF_6_] for more than 5 months. We investigated the tribological and thermal conductivity (TC) properties of the novel Au/[Bmim][PF_6_] nanofluids, and two major strategies are pursued in our studies: (1) the effects of the stability of nanofluids on their properties, and (2) the improvements of properties of [Bmim][PF_6_] induced by the introduction of low amount of Au NPs.

[Bmim][PF_6_] has been used as high performance lubricant since 2001 [20]. The nanomaterials and ILs have both been widely used as effective additives for base lubricants in the past decade [21,22], whereas the research on soft metal as additives of base ILs has not been developed yet. Therefore, the tribological properties of the Au/[Bmim][PF_6_] nanofluids with changeable stabilities were detailedly evaluated in our present work. Due to their potential applications as next generation heat transfer fluids, the TC of Au nanofluids has been studied as a function of temperature and Au NP content [16-18]. Patel et al. [16] found the temperature-dependent TC of Au/water nanofluids were greatly enhanced especially at high temperature, whereas Putnam et al. [17] and Shalkevich et al. [18] did not find this phenomenon and the TC of Au/ethanol, Au/methanol, and Au/water nanofluids were no obvious enhancements in their investigation under low temperature (≤40°C). The experimental differences of Au nanofluids and the controversy on whether the Brownian motion of nanoparticles is an important heat transfer mechanism of the nanofluids or not are always existent. Herein, we first measured the TC of Au/[Bmim][PF_6_] nanofluids using a transient hot-wire method as a function of temperature (33 to 81°C) and Au NP amount. The work conducted here is hopeful to supply experimental support and theoretical explanation on heat transfer mechanism in nanofluids.

## Experimental section

### Materials

[Bmim][PF_6_] with high purity was synthesized in our laboratory according to Ref. [23] with several small modifications. **Chloroauric acid tetrahydrate (HAuCl_4_·4H_2_O, 99.7%), hexadecyl trimethyl ammonium bromide (CTABr, 99%), and 1-Methylimidazole (98%) were purchased from Shanghai Sinopharm Chemical Regent Co., Ltd (China), 1-Methylimidazole was distilled under vacuum before used. Sodium borohydride (NaBH_4_, 98%), dichloromethane (99.5%), and anhydrous ethanol (99.7%) obtained from Tianjin Chemical Regent Co., Ltd (China) were used as received**.

### Nanofluid synthesis

The experimental parameters and stabilities of different samples are detailedly shown in Table 1. Typically, 0.03 mmol of NaBH_4 _was dissolved in 1.5 ml of [Bmim][PF_6_] by stirring and the resulting solution was kept standing for 12 h in room temperature before use. Subsequently, this solution was added into 1.5 ml HAuCl_4_·4H_2_O (2 mM) of [Bmim][PF_6_] solution containing CTABr (10 mM) under stirring at room temperature for 1/2 h, and then the sample 4 in Table 1 was obtained. The processes for synthesizing other samples are similar but the experimental parameters are varied, as shown in Table 1. The Au NPs using for characterization were collected from the sample 4 by centrifugation because the aggregation of Au NPs occurred after adding massive dichloromethane. Then, the obtained Au NPs were thoroughly washed with dichloromethane (six times) and anhydrous ethanol (three times), and dried overnight in a vacuum at 60°C.

**Table 1:** The experimental parameters and stabilities of different samples.

Sample no.	Solvent	HAuCl_4 _(mM)	Stabilizer/Au (mol/mol)	NaBH_4_/Au (mol/mol)	Stability
1	[Bmim][PF_6_]				
2	[Bmim][PF_6_]	1		10	≤2 days
3 [19]	[Bmim][PF_6_]	1	5 (1-Methylimidazole)	10	2 weeks
4	[Bmim][PF_6_]	1	5 (CTABr)	10	More than 5 months
5	[Bmim][PF_6_]	2	5 (CTABr)	10	1 week
6	[Bmim][PF_6_]	3	3 (CTABr)	10	≤4 days
7	[Bmim][PF_6_]	4	3 (CTABr)	10	≤4 days

### Characterization and property measurements

Surface Plansmon Resonance (SPR) spectra were recorded on a U-3010 UV-visible spectrometer using a quartz cell of 1 cm path length. Fourier transformation infrared (FT-IR) spectra were recorded on a **Bruker **IFS 66v/S FTIR spectrometer using the KBr disk method. X-ray photoelectron spectroscopy (XPS) analysis was obtained on a PHI-5702 multifunctional XPS. Transmission electron microscopy (TEM) analysis was conducted on a JEM-2010 transmission electron microscope at 200 kV. To prepare sample of TEM, a drop of sample 4 solution was placed on a holey-carbon coated Cu TEM grid (200 mesh). Then, the grid was rinsed with dichloromethane and dried under room temperature. The SEM/EDS analysis was performed on a JSM-5600LV scanning electron microscope.

The tribological measurements were evaluated on an Optimol SRV-IV oscillating friction and wear tester in a ball-on-disc contact configuration. The upper test piece is ϕ 10 mm GCr15 bearing steel (AISI-52100) ball, and the lower test piece is ϕ 24.00 × 7.88 mm GCr15 bearing steel (AISI-52100) flat disc. All the tests were conducted at the frequency of 25 Hz, amplitude of 1 mm, and 30 min of test duration. Prior to the friction and wear test, two drops of the sample were introduced to the ball-disc contact area. The friction coefficient curve was recorded automatically with a chart attached to the SRV-IV test rig. The wear volumes were conducted by a MicroXAM 3 D surface profilometer (ADE Phase-Shift).

Thermal conductivity of the suspension was measured using **a Decagon **KD2 pro thermometer. The KD2 is based on transient hot wire method having a probe of length 6 cm and diameter 0.13 cm. This probe integrates in the interior, a heating element and a thermoresistor, which is connected to a microprocessor for controlling as well as conducting measurements. The KD2 was calibrated using distilled water before use. In order to study the temperature effect on TC of nanofluids, a thermostat bath was used, which maintained temperature within the range of ±0.1°C. Five measurements were taken at each temperature to ensure uncertainty in the measurement within ±5%.

## Results and discussion

### Characterization and stabilization mechanism

**Figure 1:**
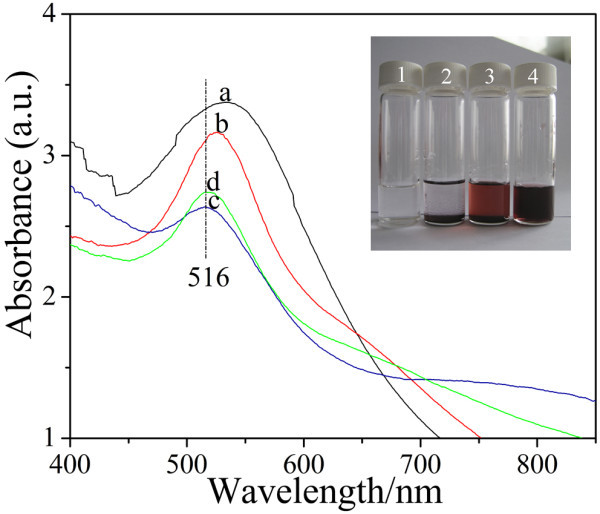
**SPR spectra of the samples (a) 2, (b) 3, (c) 4 in Table 1 after preparation and (d) the sample 4 keeping still for a month**. The *inset *is the photograph of samples 1, 2, 3, and 4 standing for a month at room temperature.

Figure 1 shows the SPR spectra of various samples. The three feature SPR absorption peaks between 510 and 550 nm in Figure 1a, b, c indicates that spherical Au NPs with different diameters and stabilities were successfully synthesized in the samples 2, 3, and 4 in Table 1. Moreover, the SPR absorption peak of Figure 1d exhibits no shift compared to that of Figure 1c, demonstrating that no aggregation occurs in sample 4 of Table 1 during a month. The photograph of various samples (the inset in Figure 1) after standing for a month shows that the complete, partial, and none sedimentation occurs in samples 2, 3, and 4 of Table 1, respectively, which also verifies the high stability of sample 4. Then, we mainly characterized the Au NPs collected from sample 4 by centrifugation in the following sections in order to disclosure the stabilization mode of Au NPs in the existence of CTABr.

**Figure 2:**
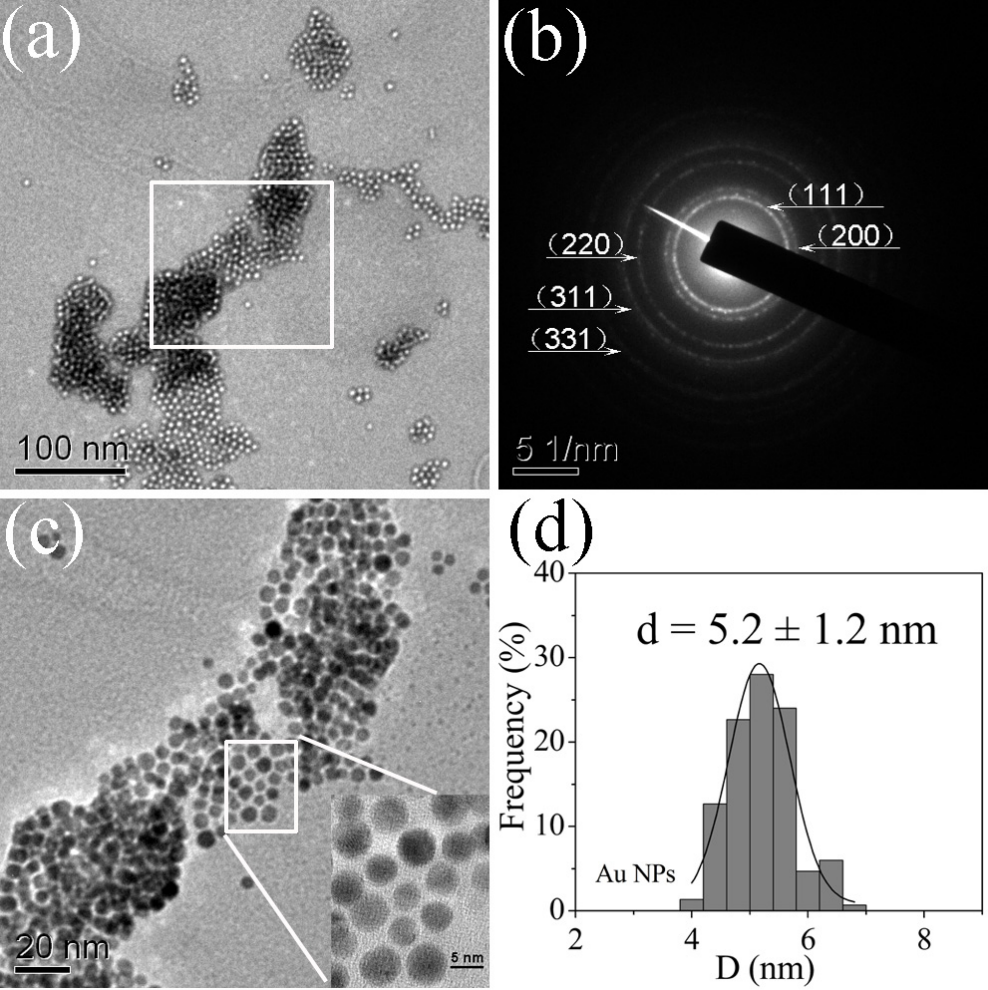
**TEM images with (a) low- and (c) high-magnification, (b) SAED pattern and (d) the size distribution of synthesized Au NPs in sample 4**.

Figure 2 shows the TEM images, the selected area electron diffraction (SAED) pattern and size distribution of Au NPs obtained from sample 4. Some extent self-assembly of spherical Au NPs of 5.2 ± 1.2 nm in diameter can be observed from Figure 2a, c, and the histogram for the size distribution of Au NPs shown in Figure 2d was obtained by counting more than 150 Au NPs. The dark place in Figure 2a can be attributed to overlap of multilayer Au NPs, whereas white place belongs to monolayer Au NPs which may be modified by CTABr. Figure 2c with high-magnification shows the region marked out in Figure 1a and verifies the conclusions mentioned from Figure 2a. The SAED pattern, as shown in Figure 2b, indicates the crystallinity of synthesized Au NPs belongs to face-centered cubic (fcc) structure. The diffraction rings corresponding to (111), (200), (220), (311), and (331) crystal planes have been marked out, respectively.

**Figure 3:**
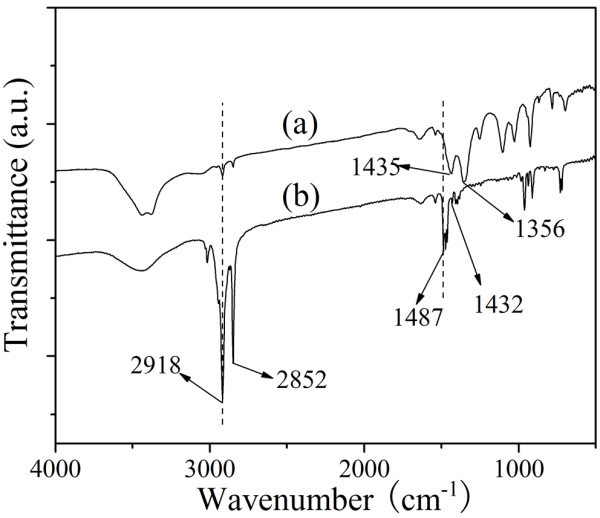
**The FT-IR spectra of (a) Au NPs obtained from sample 4 and (b) CTABr**.

**Figure 4:**
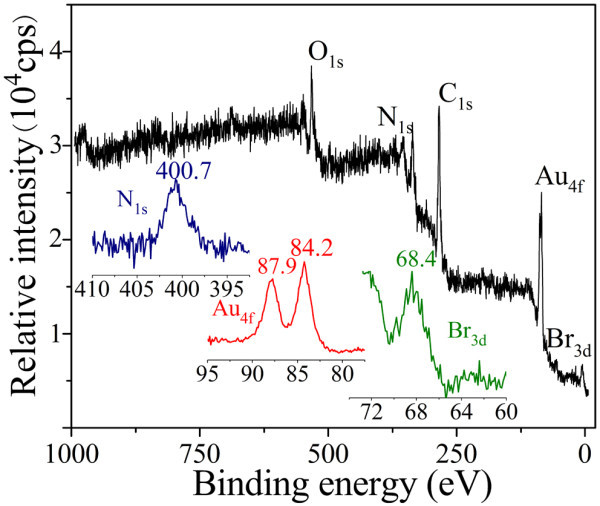
**The XPS spectrum of Au NPs modified by CTABr**. *Insets*: the N 1 s (*left*), Au 4f doublet (*middle*), and Br 3 d (*right*).

The FT-IR spectra of Au NPs and CTABr are shown in Figure 3. The C-H symmetric and asymmetric stretching vibrations of CTABr lie at 2918 and 2852 cm^-1 ^as well as those of Au NPs, indicating the CTABr molecules adsorb on Au NPs. The feature peaks at 1487 and 1432 cm^-1 ^in the spectrum of CTABr are attributed to asymmetric and symmetric C-H scissoring vibrations of CH_3_-N^+ ^moiety. They shift to 1435 and 1356 cm^-1 ^in the spectrum of Au NPs, indicating the CTABr molecules are bound to Au NPs with their headgroups. Figure 4 shows the XPS spectra of Au NPs modified by CTABr. The Au 4f_7/2 _peak appears at a binding energy of 84.2 eV and Au 4f_5/2 _peak appears at a binding energy of 87.9 eV, which indicates the formation of metallic gold [24]. The appearance of N 1 s peak (400.7 eV) and Br 3 d peak (68.4 eV) verifies the attachment of CTABr molecules on Au NPs.

**Figure 5:**
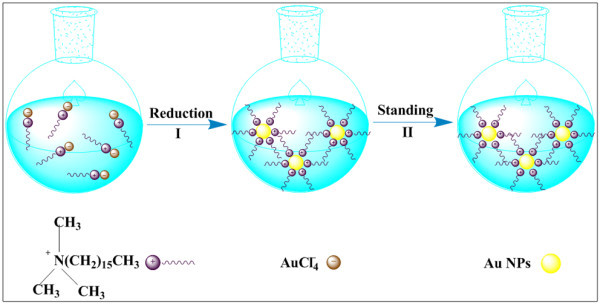
**The preparation process and stabilization mechanism of the sample 4**. I: the AuCl_4_^- ^was reduced by sodium borohydride (NaBH_4_) and Au NPs modified by CTABr were quickly obtained; II: after standing for more than 5 months, the modified Au NPs could still exhibit ultrahigh stability due to the electrostatic repulsions and steric hindrances between different Au NPs.

Based on characterization of Au NPs, the preparation process and stabilization mechanism of the sample 4 are shown in Figure 5. First, the NaBH_4 _reduced the AuCl_4_^- ^into Au NPs quickly and effectively. CTABr molecules specifically adsorbed on the Au NPs can form surface ion pairs through the attachment of Br^- ^ions to the Au surfaces and the electrostatic interactions between the cationic CTABr headgroups and the Br^- ^layer, which has been verified in a two-phase system [25]. Then, the Au NPs modified by CTABr dispersed in ILs possessed ultrahigh stability due to the electrostatic repulsions and steric hindrances among different Au NPs. Thus, the sample 4 can keep stable and homogeneous after standing for more than 5 months. While the partial aggregation of samples 5, 6, and 7 of Table 1 within 1 week indicates that this process cannot make high concentration Au nanofluids stable owing to the low solubility of CTABr in [Bmim][PF_6_].

### Tribological properties

**Figure 6:**
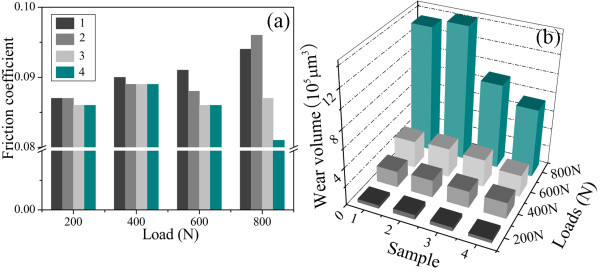
**The friction coefficients (a) and wear volumes (b) of steel discs lubricated by samples 1, 2, 3, and 4 under various loads**.

**Figure 7:**
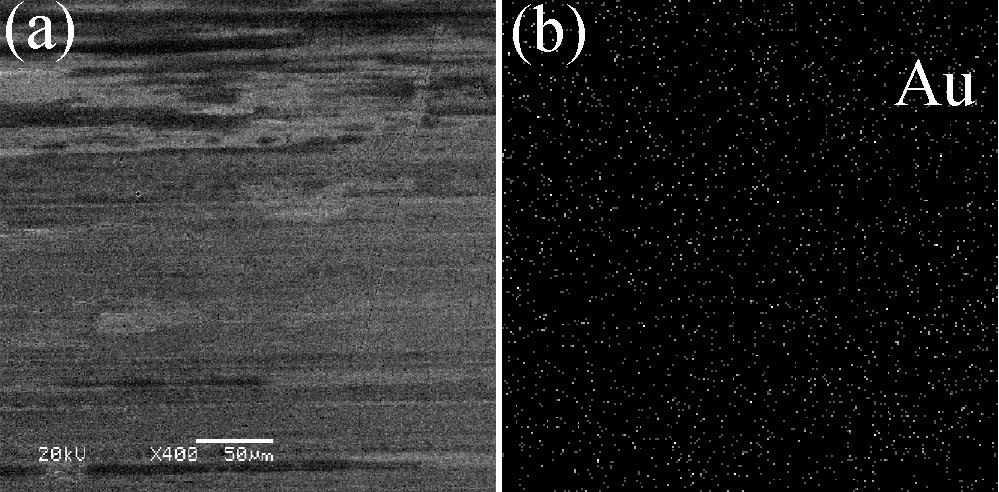
**SEM (a) and EDS (b) images of the worn surface lubricated by the sample 4 under 800 N and the element distribution of Au**.

Figure 6 shows the friction coefficients and wear volumes of steel discs lubricated by samples 1, 2, 3, and 4 under loads in range of 200 to 800 N. Under low loads of 200 and 400 N, the friction coefficients lubricated by the Au/[Bmim][PF_6_] nanofluids (samples 2, 3, and 4 of Table 1) are slightly lower than that of pure [Bmim][PF_6_] (sample 1 of Table 1), exhibiting slight friction-reduction properties. However, there are no obvious reduction but even slight increment for the wear volumes lubricated by the nanofluids compared with pure [Bmim][PF_6_], which can be attributed to the occurrence of adhesive wear because the gold is softer than steel. While under high loads of 600 and 800 N, the Au NPs during friction process may first fill up the micro-gap of rubbing surface and deposit there to form a self-assembly thin film, which could provide protection for the surface from serious abrasive wear [22]. It is confirmed by SEM and EDS images of the worn surface lubricated by the sample 4 under 800 N, as shown in Figure 7. In Figure 7, it can be observed that the worn surface is smooth and the Au element homogeneously distributes on the rubbing surface, verifying that no abrasive wear occurs and a protective thin film composed of Au NPs forms during friction process. Therefore, the stable nanofluids (samples 3 and 4 of Table 1) are helpful to form a self-assembly metal film and exhibit excellent fiction-reduction and anti-wear ability when they are under the load of 600 or 800 N. For example, the friction coefficient and wear volume of sample 4 are 13.8 and 45.4% lower than those of sample 1 in Table 1 under 800 N. On the contrary, the unstable Au NPs dispersion of sample 2 in Table 1 may bring about ruleless aggregation but not self-assemble behavior of Au NPs during friction so as to result in destruction of the layer structure film of [Bmim][PF_6_] [26] on the specimen and serious abrasive wear, leading to high friction coefficient and large wear volume.

**Figure 8:**
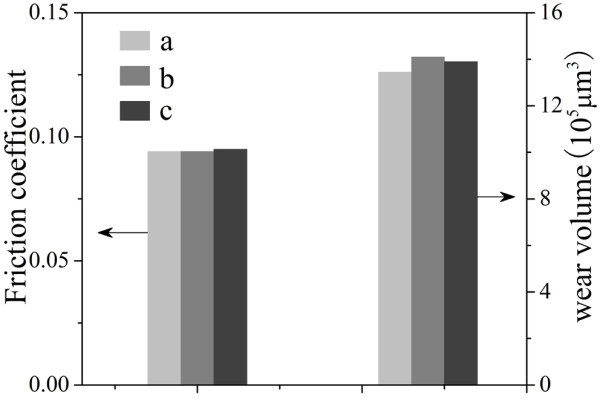
**The friction coefficients and wear volumes of discs lubricated by (a) pure [Bmim][PF_6_], (b) [Bmim][PF_6_] containing 1-methylimidazole (5 mmol), and (c) [Bmim][PF_6_] containing CTABr (5 mmol) under 800 N**.

Figure 8 shows the friction coefficients and wear volumes of discs lubricated by pure [Bmim][PF_6_], [Bmim][PF_6_] containing 1-methylimidazole (5 mmol), and [Bmim][PF_6_] containing CTABr (5 mmol) under 800 N. The addition of small amount stabilizer (1-Methylimidazole or CTABr) into [Bmim][PF_6_] introduces slight increments of friction coefficient and wear volume in the tribological measurements, indicating that the stabilizers used in the nanofluids have slightly negative effects on the tribological properties of [Bmim][PF_6_]. Then, it is not difficult to understand that the much better tribological properties of the Au/[Bmim][PF_6_] nanofluids (samples 3 and 4 of Table 1) must be attributed to the existence of stable Au NPs but not 1-methylimidazole or CTABr.

**Figure 9:**
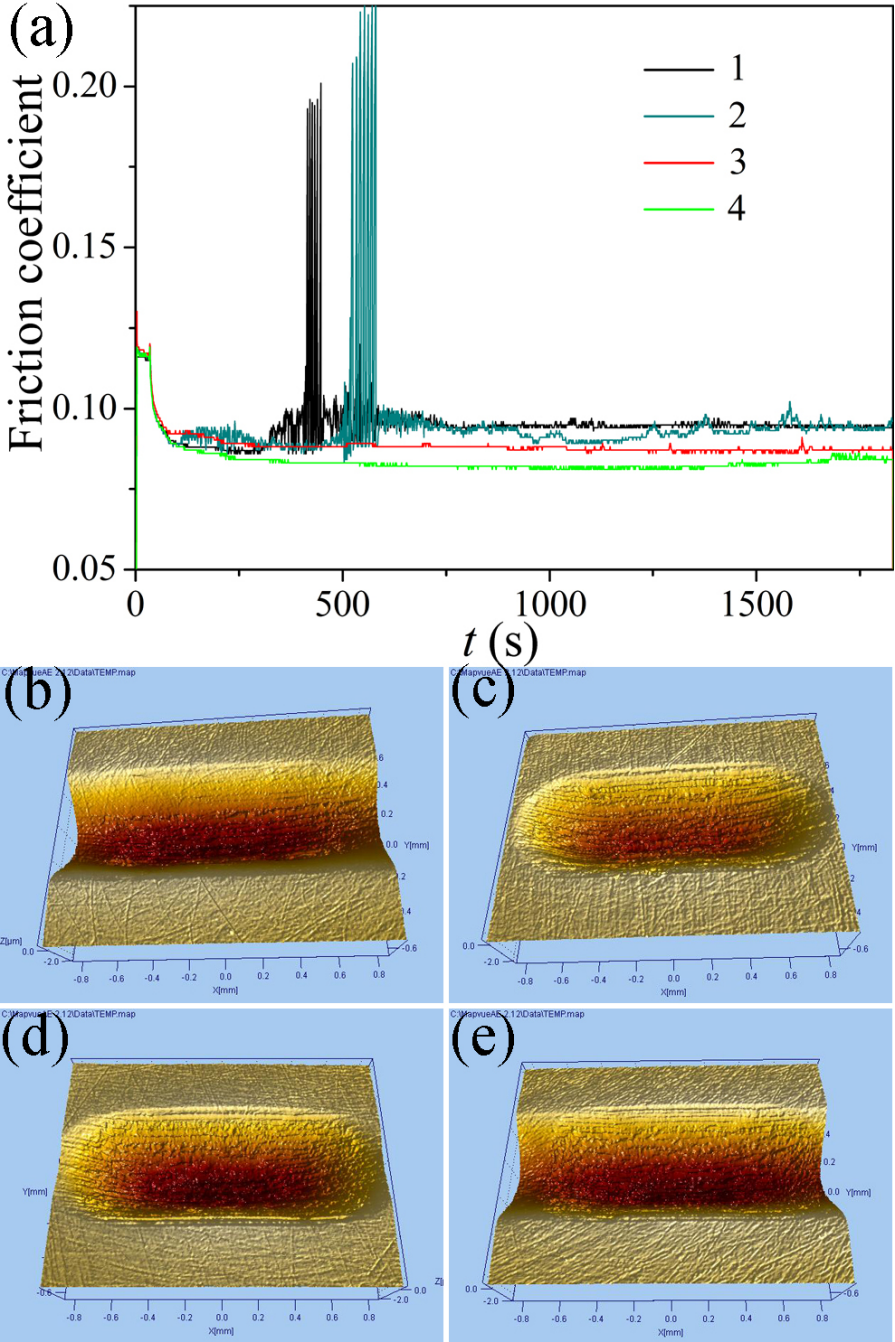
**Friction coefficient curves lubricated by various samples as a function of time under 800 N (a), and 3 D images of the worn surfaces lubricated by samples 1 (b), 2 (c), 3 (d), and 4 (e) under 800 N**.

To further verify that the stable Au/[Bmim][PF_6_] nanofluids have much better tribological properties under high loads, the corresponding friction coefficient curves under 800 N as a function of time and the three dimension (3D) images of worn surfaces lubricated by all four samples were measured, as shown in Figure 9. The friction coefficients of samples 1 and 2 in Table 1 fiercely fluctuate in running-in period during test in Figure 9a, indicating the existence of the serious abrasive wear. This phenomenon is corresponding to their 3 D images of worn surfaces shown in Figure 9b, c, which exhibit large wear volumes and serious abrasion. On the contrary, the friction coefficient curves of samples 3 and 4 in Table 1 are lower and smoother than those of samples 1 and 2 in Table 1, showing obvious friction-reduction properties. Accordingly, their 3 D images of worn surfaces in Figure 9d, e show smaller wear volumes and slight abrasion compared to those of samples 1 and 2, exhibiting favorable anti-wear properties.

**Figure 10:**
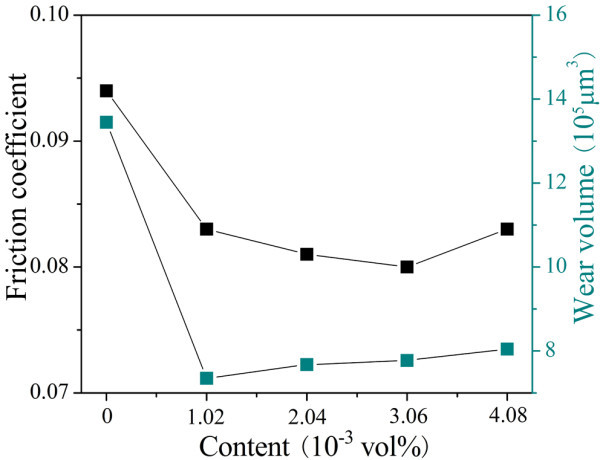
**The friction coefficients and wear volumes of discs lubricated by Au/[Bmim][PF_6_] nanofluids containing CTABr with various concentrations under 800 N**.

We assume the HAuCl_4 _was completely reduced by access NaBH_4_. Then, the volumetric fraction of the samples 4, 5, 6, and 7 in Table 1 are 1.02 × 10^-3^, 2.04 × 10^-3^, 3.06 × 10^-3^, 4.08 × 10^-3^%, respectively. The friction coefficients and wear volumes of discs lubricated by Au/[Bmim][PF_6_] nanofluids using CTABr as stabilizer with various volumetric fraction (vol.%) under 800 N are shown in Figure 10. It has been found that the addition of low concentration Au NPs modified by CTABr greatly improves the tribological properties of basic lubricant ([Bmim][PF_6_]). And the effects of concentration on tribological properties of nanofluids are not obvious. In comparison with concentration, the stability of the Au NPs used as additives is of key importance in improving the tribological properties of pure [Bmim][PF_6_].

### Thermal conductivity

The volumetric fraction of Au NPs of sample 4 in Table 1 with ultrahigh stability is about 1.02 × 10^-3^% as mentioned above. The Au/[Bmim][PF_6_] nanofluids with concentrations of 2.55 × 10^-4 ^and 5.1 × 10^-4^% were also fabricated by diluting the sample 4 before the TC measurements. Compared with traditional heat transfer oil, the [Bmim][PF_6_] possesses slightly higher TC, much higher thermal stability, lower volatility, and nonflammability, which make it be a potential high-temperature heat transfer fluid in the future. However, the poor TC of [Bmim][PF_6_] [27] still needs to be enhanced. Moreover, the temperature is a key factor for the investigation of heat transfer mechanism in nanofluids. Thus, the TC of Au/[Bmim][PF_6_] nanofluids was measured as a function of temperature in our following work.

**Figure 11:**
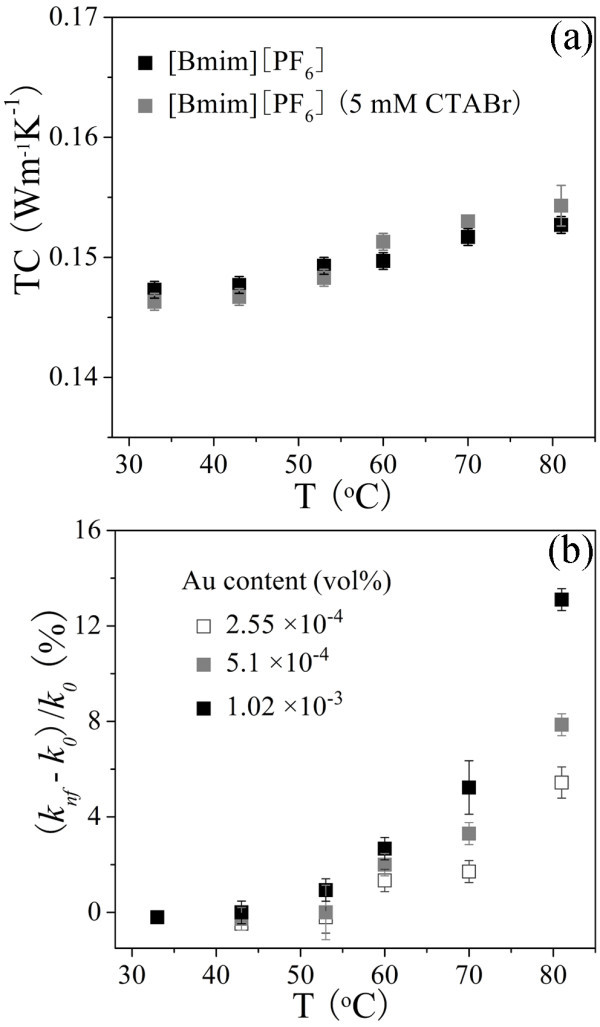
**The TC of [Bmim][PF_6_] and [Bmim][PF_6_] containing CTABr (5 mmol) (a), and the TC enhancement of Au/[Bmim][PF_6_] nanofluids (b) with various concentrations varying with temperature**.

Figure 11 shows the TC of [Bmim][PF_6_] and [Bmim][PF_6_] containing CTABr (5 mM) and the TC enhancements of Au/[Bmim][PF_6_] nanofluids defined as (*k*_nf _- *k*_0_)/*k*_0 _(%) with various concentrations in temperature range of 33 to 81°C, where *k*_nf _and *k*_0 _is TC of the nanofluids and [Bmim][PF_6_] at various temperatures, respectively. The TC of [Bmim][PF_6_] and [Bmim][PF_6_] containing CTABr (5 mM) in Figure 11a is both slightly temperature-dependent and the later is no remarkable differences compared with the former, indicating the low amount of CTABr has no obvious effects on the TC of [Bmim][PF_6_]. Therefore, the effects on the TC of base liquid induced by CTABr are omitted in the following discussion on the TC enhancements of the nanofluids. The TC enhancements of nanofluids in Figure 11b increases slightly at low temperatures (≤53°C) but sharply at high temperatures (≥60°C), exhibiting non-linear increment as a function of temperature and the remarkable effect of stable Au NPs on the TC of base liquid especially at high temperatures. The TC of the nanofluid (1.02 × 10^-3^%) at 81°C is 13.1% higher than that of base liquid, indicating the addition of low concentration of stable Au NPs can greatly improve the thermal properties of [Bmim][PF_6_] under high temperature.

**Figure 12:**
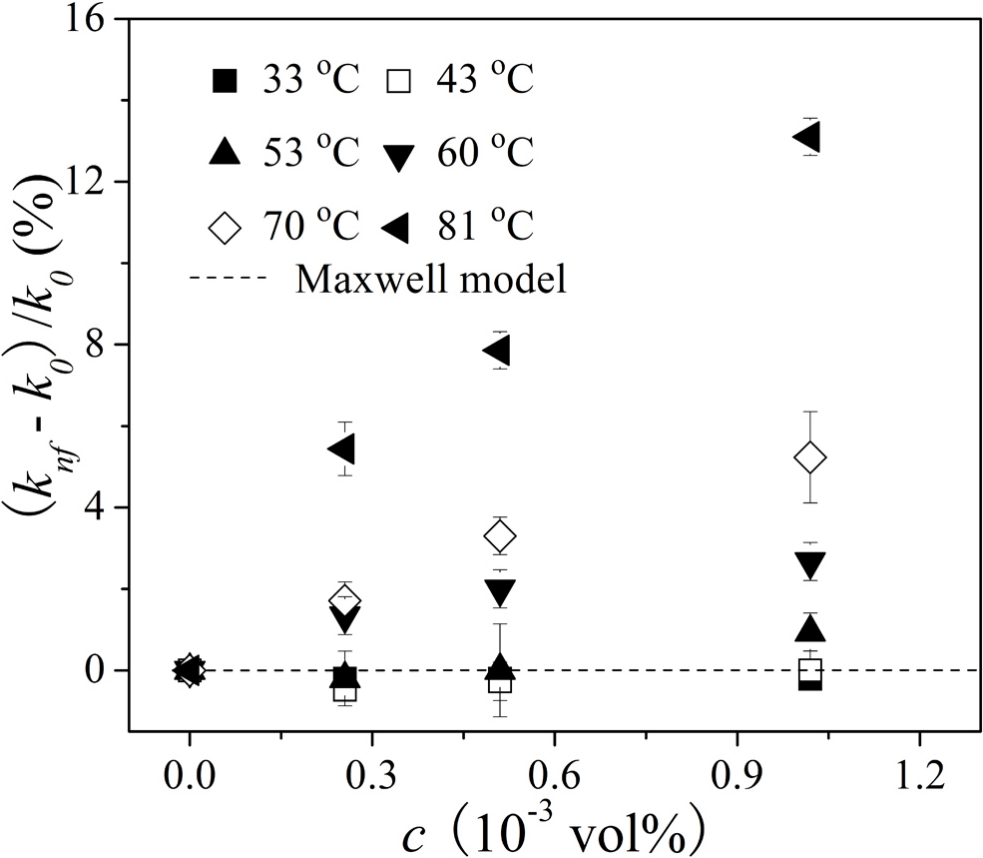
**The TC enhancement of Au/[Bmim][PF_6_] nanofluids as a function of concentration under various temperatures**. The *dashed line *corresponds to the Maxwell effective medium theory.

The relationship between the TC enhancement and the concentration under various temperatures is illustrated in Figure 12. The Maxwell effective medium theory [28] which can be simplified to *k*_nf _= (1 + 3φ) *k*_0 _when *k*_0 _< <*k*_p _was also drawn in Figure 12, where *k*_p _is the TC of the nanoparticles and φ is the volumetric fraction of the nanofluid. The differences of the TC enhancement are negligible when the temperature is lower than 53°C and could be predicted by the Maxwell effective medium theory very well. However, the TC enhancement of the Au nanofluids gradually exhibits non-linear increment with the increment of volumetric fraction when the temperature is higher than 60°C and is much higher than the estimation of the Maxwell model. Moreover, the temperature is higher, the TC enhancement rate is sharper.

The TC enhancement of nanofluids showing a strong sensitivity to the temperature was also found by some other researchers [16,29-33]. Among the various proposed mechanisms of ballistic heat transfer of nanoparticles, nano-layers of liquid molecules around nanoparticles, clustering of nanoparticles, and the Brownian motion of nanoparticles for the anomalously enhanced TC of nanofluids compared to that of base liquids [34], the micro-convection caused by the Brownian motion of nanoparticles is the most reliable explanation for low concentration nanofluids [35]. In our experiments, these Au nanofluids with low concentrations exhibit little enhancements under low temperature but obvious enhancements under high temperature. The relationship between the concentration and the TC enhancement is negligibly and sharply relative under low and high temperature, respectively. All these phenomena verify that the micro-convection caused by the Brownian motion of nanoparticles plays the most important role in the TC enhancement of Au nanofluids compared with other heat transfer mechanisms of the nanofluids. Then, it is not difficult to understand the results in our work. The viscosity of base liquids and temperature are two factors influencing the Brownian motion of Au NPs. The increase of temperature would cause large viscosity reduction of [Bmim][PF_6_] with a large viscosity-temperature exponent and aggravate the Brownian motion of Au NPs. These changes of Au/[Bmim][PF_6_] nanofluids with the increase of temperature can be the reason why the TC of nanofluids is conspicuously temperature-dependent and greatly enhanced especially at high temperatures.

## Conclusions

The Au/[Bmim][PF_6_] nanofluids with changeable stabilities were synthesized by a facile Brust-Schiffrin method at room temperature. The reliable encapsulation mechanism was proposed for the nanofluids with ultrahigh stability by UV-visible, TEM, FT-IR, and XPS characterizations of Au NPs. The electrostatic repulsion and steric hindrance between Au NPs modified by CTABr make the Au NPs keep stable in [Bmim][PF_6_] for a long time. In comparison with pure [Bmim][PF_6_], the stable nanofluids exhibited excellent friction-reduction and anti-wear properties even if the addition concentration of Au NPs was very low, which indicated that the stability of the nanofluids is of key importance. Moreover, the TC of stable Au/[Bmim][PF_6_] nanofluids were also measured as a function of temperature. The TC of nanofluids is sharply temperature-dependent and greatly enhanced compared to that of pure [Bmim][PF_6_], which can be attributed to the micro-convection caused by the Brownian motion of Au NPs. To sum up, the additions of stable Au NPs with low concentrations can greatly improve the physicochemical properties of [Bmim][PF_6_]. Therefore, more Au/IL nanofluids with high stability need to be prepared and their other properties also need to be exploited in the future, which might broaden their potential applications in the fields of photonics, optoelectronics, sensor, catalysts, lubricants, heat transfer liquids, information storage, and medicine.

## Competing interests

The authors declare that they have no competing interests.

## Authors' contributions

BW did the synthetic and characteristic job in this manuscript. XW, WL, and JH gave the advice and guide for the experimental section and edited the manuscript. All authors read and approved the final manuscript.
